# Interparticle Ion Migration in Cesium Lead Mixed-Halide Perovskite Nanocrystal Superlattices

**DOI:** 10.1021/acs.nanolett.6c00452

**Published:** 2026-03-27

**Authors:** Ata Bozkurt, Jonas L. Hiller, Robert Thalwitzer, Mario Martin, Ivan Musil, Elke Nadler, Ross Ewan Carter, Martin Eberle, Jonas Haas, Ivan A. Zaluzhnyy, Jannik C. Meyer, Frank Schreiber, Marcus Scheele

**Affiliations:** † Institute of Physical and Theoretical Chemistry, 9188 University of Tübingen, Auf der Morgenstelle 18, 72076 Tübingen, Germany; ‡ Institute of Applied Physics, 9188 University of Tübingen, Auf der Morgenstelle 10, 72076 Tübingen, Germany; § NMI Natural and Medical Sciences Institute at the University of Tübingen, Markwiesenstraße 55, 72770 Reutlingen, Germany

**Keywords:** Superlattice, SEM, perovskite, ion migration, micromanipulation, confocal microscopy, EDX

## Abstract

Lead mixed-halide perovskite nanocrystals offer exceptional optical properties but suffer from ionic instability and ion migration under external stimuli, challenging their integration into devices. While such effects have been well studied in individual NCs and films, their impact on nanocrystal assemblies remains less understood. Here, we investigate the effect of strong external electric fields on self-assembled CsPbBr_2.4_Cl_0.6_ nanocrystal superlattices. By positioning individual superlattices between micrometer-sized capacitor plates, we analyze field-induced changes in photoluminescence, elemental composition, and morphology. We observe position-dependent changes in emission energy correlated with halide ion redistribution, revealed by energy-dispersive X-ray analysis, resulting from a nonuniform electric field across the superlattice, and supported by finite-element simulations. In situ mass spectrometry detects bromide sublimation, suggesting a combination of inter- and intraparticle halide diffusion. Irreversibility of photoluminescence and morphological changes further support a field-driven reorganization. These findings reveal responses of CsPbBr_2.4_Cl_0.6_ superlattices subject to external electric fields, relevant for their implementation in optoelectronic applications.

## Graphical abstract

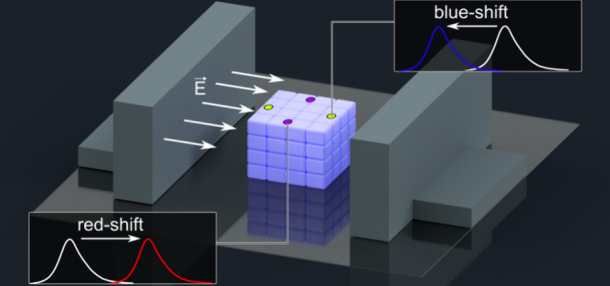

Colloidal lead halide perovskites (LHPs) nanocrystals (NCs), have emerged as promising materials in material science and nanotechnology research thanks to their remarkable optoelectronic properties such as near unity photoluminescence quantum yields (PLQY), exceptionally narrow emission line widths, and high color purity.^1–4^ These defining optical characteristics render them attractive for applications in photovoltaics, photodetection and light-emitting devices.^5–16^ Beyond the properties of individual LHP NCs, many studies have focused on their assembly into long-range ordered arrays known as superlattices (SLs),^17–20^ using different self-assembly processes such as solvent evaporation^18,19,21–24^ or two-layer phase diffusion with an antisolvent.^22^ These processes not only remove excess ligands and allow size focusing of the NC ensemble, but also direct its self-assembly into SLs, where coherent excitonic coupling enables superfluorescence.^22,25,26^ A recent study compared the Young’s modulus between CsPbX_3_ SLs fabricated via antisolvent-induced phase diffusion and solvent evaporation, showing that SLs crystallized via the former method are more mechanically robust, enabling their precise manipulation and integration into microdevices.^22^ A distinct, yet often problematic characteristic of the LHP lattice is its large ionic conductivity, enabling strong ion diffusion, for instance upon heating, photoexcitation, or due to an external electric field.^27–33^ A direct and widely studied consequence of this ionic lability is the phenomenon of halide phase segregation in mixed-halide perovskites, such as CsPb­(Br_*x*_I_1–x_)_3_. Under a persistent stimulus, an initially homogeneous, mixed-halide crystal forms distinct domains enriched in different halides.^31–33^ This segregation fundamentally alters the bandgap of the LHP and the optical properties, presenting a serious challenge for applications that demand stable and precisely tunable color output.^32^ In studies on isolated or assembled CsPb­(Br_*x*_I_1–x_)_3_ NCs, prolonged illumination can lead to a photoinduced blue-shift in emission. This has been attributed to the preferential breaking of weaker Pb–I bonds and the subsequent migration and emission of iodide ions from the nanocrystal, leaving behind a more bromine-rich, higher-bandgap material.^33,34^ Brennan et al. demonstrated that prolonged illumination of the LHP SL does not lead to reversible segregation, but rather to an irreversible compositional transformation.^32^ Zhang et al. investigated the intrinsic effects of an external electric field on mixed-halide perovskites thin films. They demonstrated that a reversible photoluminescence blue-shift in CsPbBr_1.2_I_1.8_ nanocrystal thin films is induced solely by an applied electrical bias in the absence of illumination.^33^ This observation led to the proposal that the local electric field is the universal trigger for the initial breaking of ionic bonds, which is the prerequisite for the entire ion migration cascade. In this model, photogenerated carriers become trapped at surfaces or defects, which creates a local field to initiate ion migration.^33^

From this, a critical knowledge gap emerges. Although the effects of light on mixed halide SLs and the general effects of electric fields on perovskite devices are understood, the direct, fundamental impact of a strong external electric field on the structural, compositional, and optical integrity of a well-defined CsPbBr_2.4_Cl_0.6_ SL has not yet been systematically investigated. In this study, we examine the impact of an externally applied electric field on the structural and optical properties of self-assembled CsPbBr_2.4_Cl_0.6_ nanocrystal superlattices obtained by an antisolvent-based two-layer diffusion process. By exploiting their high mechanical robustness, we position selected SLs inside the capacitor-plates of a prefabricated Pt microcapacitor. Using optical spectroscopy and composition characterization techniques such as elemental dispersive X-ray analysis, we examine the potential mechanisms of field-induced ion migration within the SL and correlate changes in optical emission with structural and compositional transformations. Furthermore, we test the hypothesis that a strong electric field can directly induce halide sublimation from the nanocrystal surface. This study sheds light on the ionic response mechanisms in and stability limits of mixed-halide perovskite superstructures exposed to strong electric fields, the understanding of which is crucial for their potential use in optoelectronic devices.

In this section we explain the fabrication process of the microcapacitors on a glass substrate used to generate the electric fields across the SLs in this work. Prior to substrate fabrication, large gold contact pads that are separated by 200 μm channels are deposited by optical lithography. Next, microplates at the end of two adjacent contact pads are fabricated using a gas injection system (GIS) inside a scanning electron microscope (SEM), employing an organoplatinum precursor (C_3_H_6_PtCpCH_3_) and a focused gallium-ion beam (FIB) as shown in Figure 1a and b. Height and distance between the plates can be customized according to the size of the superlattices exemplarily shown in Figure 1c. An overview of the crystal handling is illustrated in Figure 1d, where a suitable perovskite SL is selected (Figure 1e) and placed between the capacitor plates (Figure 1f) using microgrippers. We analyze the effect of an electric field on the CsPbBr_2.4_Cl_0.6_ SL by confocal laser stage-scanning microscopy under ambient conditions, mass spectrometry, and energy dispersive X-ray spectroscopy (EDX). A detailed explanation of the confocal microscope and mass-spectrometer, as well as general information about the materials and methods used, is provided in the Supporting Information (SI).

**Figure 1:**
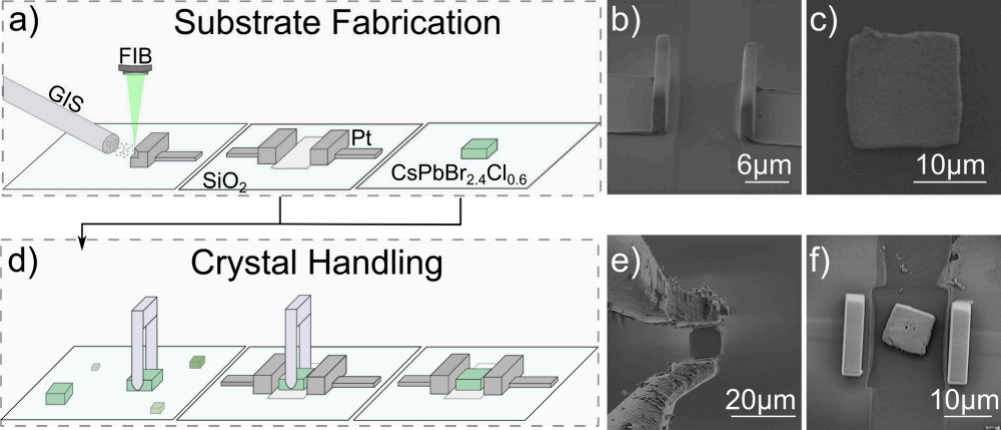
(a) Schematic illustration of the substrate fabrication process with plate capacitor and growth of SL on separate substrates. (b) SEM image of the fabricated plate capacitors on a glass substrate. (c) SEM image of a CsPbBr_2.4_Cl_0.6_ SL on a silicon wafer. (d) Schematic illustration of the crystal handling with microgrippers. (e) SEM image of the microgripper during crystal handling. (f) SL positioned inside the plate capacitor.

As a first step, we ensured that the PL across the SL initially exhibits only weak spatial fluctuations arising from local differences in its mixed-halide composition. To verify this, we recorded a spectral map of a CsPbBr_2.4_Cl_0.6_ SL prior to the application of an electric field. Figure 2a displays an intensity-weighted map of the PL peak energy where each pixel is subject to a full PL spectrum. We analyze three points along the SL and plot the underlying spectra at the respective positions in Figure 2b. The SL exhibits spatially relatively uniform PL emission in a narrow peak energy range from 2.538 eV (first quartile) to 2.543 eV (third quartile) (Figure S5 and Table S2). Reasons for the observed small heterogeneities could be local fluctuations in halide composition, strain,^36^ NC size,^22^ NC coupling,^18^ or a combination thereof.

**Figure 2:**
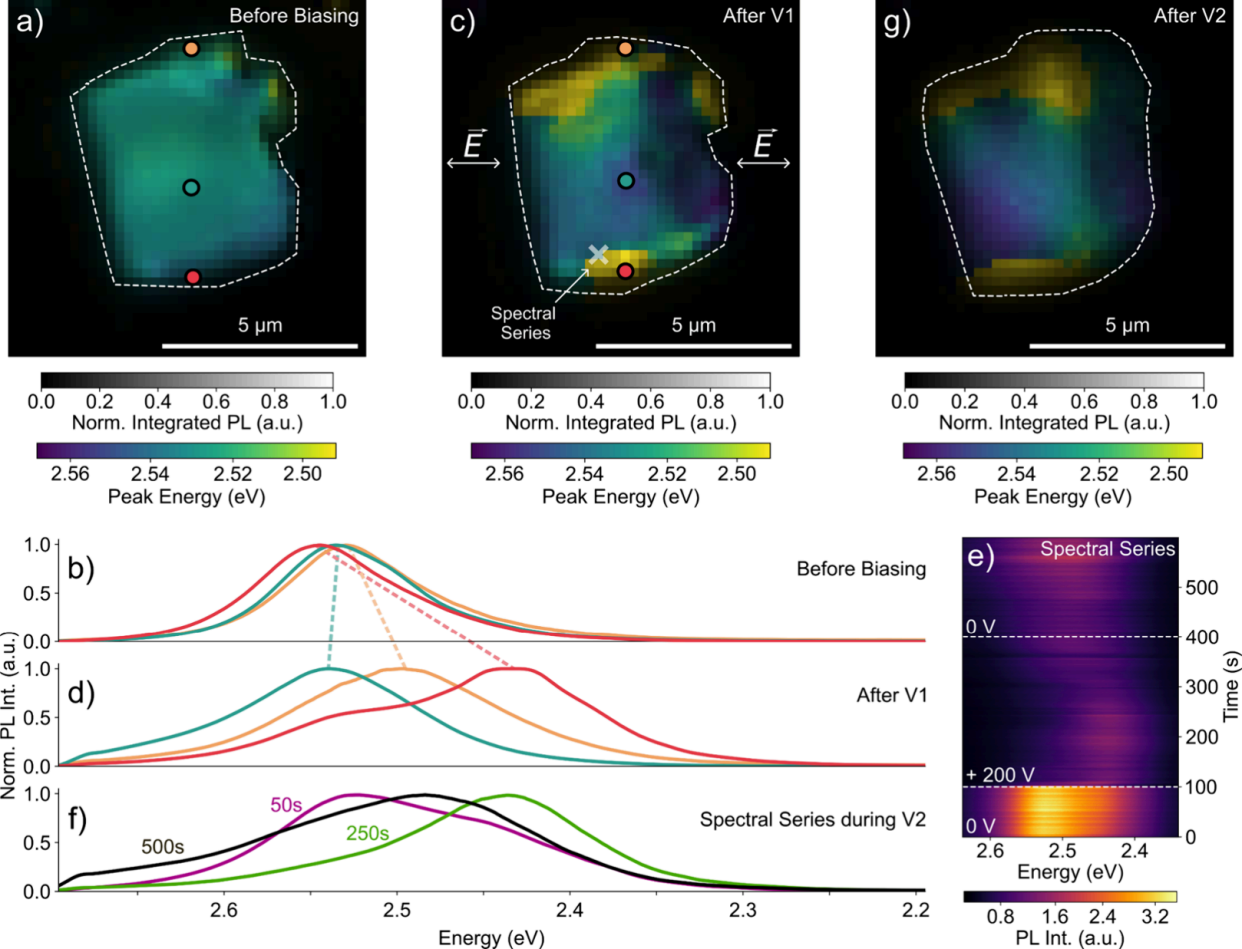
(a) Emission spectral map of a CsPbBr_2.4_Cl_0.6_ SL inside a microcapacitor with a plate-to-plate spacing of 12 μM and no bias applied (white dashed lines highlight the morphology of the SL). (b) Corresponding PL spectra at the colored dots in the SL without applied bias. (c) Emission spectral map of the CsPbBr_2.4_Cl_0.6_ SL recorded following the first biasing step (after V1). White arrows indicate the direction parallel to the electric field. (d) Corresponding PL spectrum at the colored dots in the SL. (e) Series of PL spectra recorded from the location marked by the white cross in (c). The white dashed lines indicate the time points at which the +200 V bias was turned on and off. (f) Selected PL spectra from the spectral series shown in (e). (g) Emission spectral map of the SL after the second biasing step (after V2).

In a first biasing step (V1), we then apply +200 V to the microplate capacitor for 300 s, thereby generating a strong electric field of approximately 167 kV/cm (200 V/12 μm = applied bias/plate-to-plate spacing) along the direction indicated by the arrows, and subsequently record another intensity-weighted map of the PL peak energy, displayed in Figure 2c. We observe only slight changes in the PL peak energy from the central region of the SL in response to the electric field. In contrast, there are substantial changes at the corners and along the edges of the crystal. The edges of the crystal parallel to the electric field display a red-shift of up to 130 meV. The right edge of the crystal, which is orthogonal to the direction of the electric field, exhibits opposite behavior with a slight blue-shift of 3 meV. This is further detailed in Figure 2d with PL spectra taken at the three positions indicated in Figure 2c. Most notably, position 1 (indicated by the orange dot) and 3 (indicated by the red dot), both located at the edges of the SL parallel to the field, exhibit red-shifting PL by 30 and 112 meV respectively. In contrast, position 2 (indicated by the blue dot) exhibits a slight blue-shift, by 7 meV from 2.538 to 2.545 eV.

To verify these observations, in a second biasing step (V2), we conduct a series of PL spectra at the position marked with a white cross in Figure 2c with +200 V applied for 382 s at a rate of one spectrum per second. The results are shown in Figure 2e. The PL spectra before, during, and after application of the bias are shown in Figure 2f. Resulting from the first biasing step, the initial spectra (until 100 s) already exhibit a pronounced low energy shoulder around 2.42 eV. Upon application of the bias, we observe an initially rapid shift of the emission to 2.42 eV, with most of the change occurring within 10 s, followed by a slower, gradual shift. At 250 s (150 s after the bias application) only a slight high-energy tail remains of the 2.54 eV peak. Then, while the bias is still applied, the spectrum begins to gradually shift back to 2.48 eV. The spectral dynamics are discussed in more detail in section 5 of the SI. Figure 2g shows the PL spectral map of the CsPbBr_2.4_Cl_0.6_ SL recorded following the spectral series at zero bias. The second biasing step appears to have furthered the spatial segregation in the emission energy. Changes in the morphology of the crystal can be seen by comparing the shape of the SL in Figure 2a, c, and g (white dashed lines) by using the PL intensity signal as contrast. This comparison indicates a change in the SL morphology. Occasionally, we observed that this structural change can lead to short circuiting of the capacitor and large structural disruptions of the SL (Figure S8).

We substantiate these findings by analyzing another CsPbBr_2.4_Cl_0.6_ SL shown in Figure 3. Figure 3a shows a SEM image of the SL placed between microcapacitor plates before the application of a bias. During a first biasing step (V1) we record a series of PL spectra at the position marked with a blue cross in the SEM close-up in Figure 3b. At zero bias we observe a steady PL at 2.50 eV, followed by a pronounced blue-shift to 2.64 eV following the application of a + 50 V bias (Figure 3c), generating an electric field with an approximately strength of 42 kV/cm. Selected time stamps illustrating the gradual blue-shift are plotted in terms of their PL spectra in Figure 3d.

**Figure 3:**
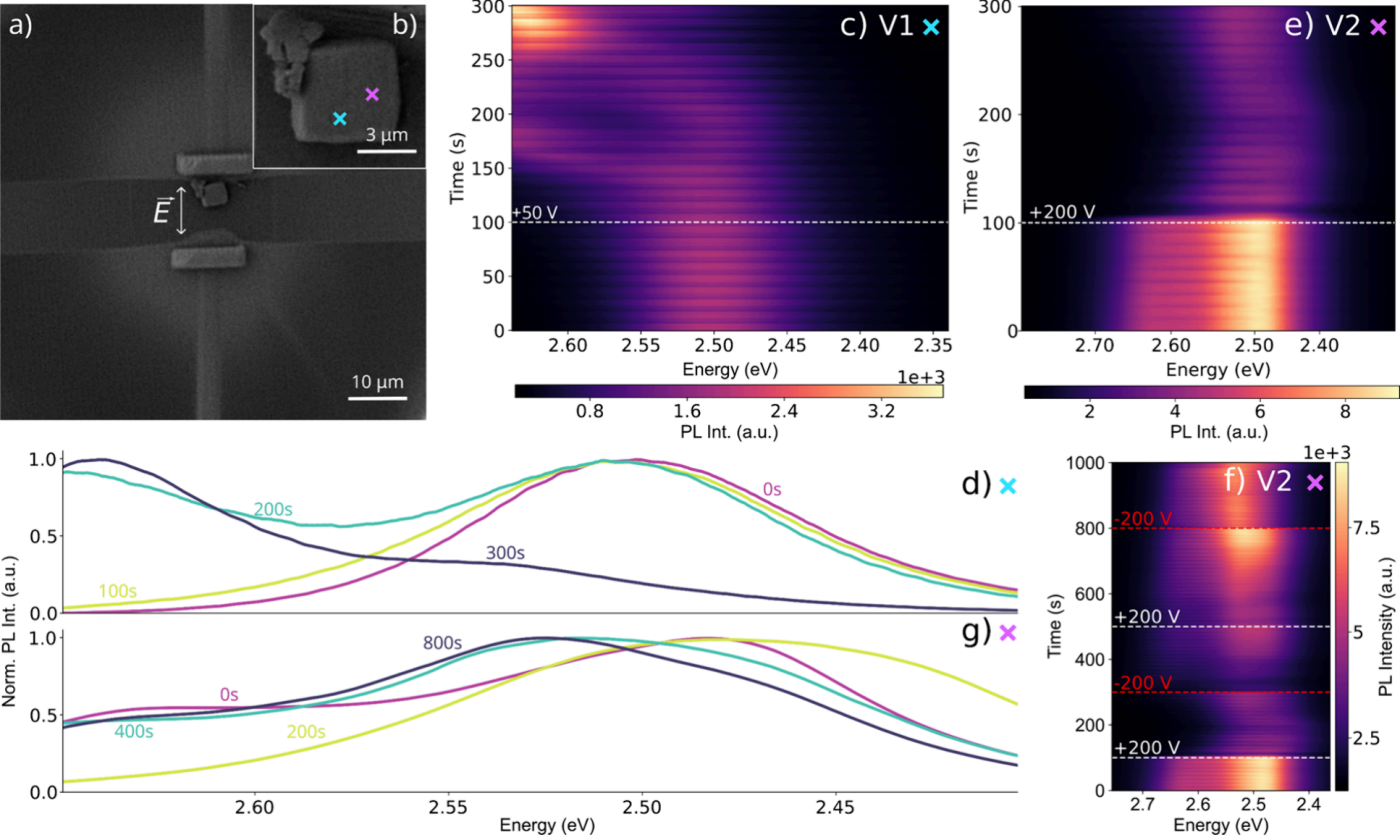
(a) SEM image of a CsPbBr_2.4_Cl_0.6_ SL placed inside a microcapacitor with a plate-to-plate spacing of 12 μM. (b) Close-up SEM image of the SL with marked positions where measurements were recorded (the color of each cross refers to the corresponding PL spectra series and its position on the SL). (c) Series of PL spectra recorded from the position marked by the blue cross in (b), with a white dashed line indicating when a +50 V bias was applied to the capacitor. (d) PL spectra from the series in (c) at selected timestamps. (e, f) Second series of PL spectra measured at the purple cross in (b), where white and red dashed lines indicate when the polarization of the 200 V bias was changed. (g) PL spectra at selected timestamps from the series shown in (f).

A second series of PL spectra is subsequently recorded from a different position on the same SL, located near an SL edge oriented parallel to the applied electric field (purple cross) during a second biasing step (V2). In this series, the polarity of the applied bias is varied between +200 V and −200 V. The first 300 s of this series are shown in Figure 3e, while the full series is presented in Figure 3f. PL spectra taken at selected time stamps in Figure 3g monitor the effect of varying the bias between +200 V and −200 V (electric field strength of approximately 167 kV/cm).

Starting at 0 V, we observe steady PL centered at 2.50 eV with a secondary PL maximum at 2.64 eV (spectrum at 0 s). At this position, under +200 V bias (spectrum at 200 s), we note a red-shift to 2.48 eV accompanied by the disappearance of the second PL peak. As apparent in Figure 3e, consistent with the spectral series shown in Figure 2e, the emission subsequently exhibits a gradual shift back toward higher energies despite the continued presence of the applied bias. Reversing the polarity of the electric field to −200 V blue-shifts the PL to 2.55 eV and invokes the recurrence of a second PL maximum at 2.64 eV (spectrum 400 s). These qualitative trends persist with repeated polarity switches. Throughout the series, the emission intensity fluctuates with each change in bias polarity resulting in a temporary intensity drop.

To obtain better insights into the composition of the CsPbBr_2.4_Cl_0.6_ superlattice and its electric-field induced changes, we display energy-dispersive X-ray (EDX) results before and after application of a 200 V bias in Figure 4. Corresponding EDX spectra can be found in the SI (Figure S10). Figure 4a–d shows element-specific EDX maps of the initial SL, while Figure 4e to 4h contain the same EDX maps of another representative SL after exposure to an electric field of approximately 167 kV/cm. In Figure 4e the direction of the electric field is visualized by the white arrow, which also applies for Figure 4f–h. While the distribution of lead (Figure 4g) seems unchanged, the chloride map (Figure 4f) shows small areas where chloride ions are undetectable. Such areas are even more pronounced for the bromide map (Figure 4e), where we find a significant area with almost no bromide (blue dashed rectangle). Conversely, the cesium map in Figure 4h shows a higher concentration of cesium ions at this exact spot (white dashed rectangle). To complement these findings, Figure 4i shows a spatially resolved PL image of the same CsPbBr_2.4_Cl_0.6_ SL. Figure 4j, k, and l depict PL spectra recorded at nine specific positions indicated in Figure 4i. Examining the PL spectra in Figure 4j, a blue-shift is evident at point 1, while points 2 and 3 exhibit a red-shift. Points 4 and 6 in Figure 4k show a red-shift, while the PL at point 5 is unchanged. Focusing on point 4 reveals a shoulder formation at 2.47 eV. Points 7 to 9 in Figure 4l all exhibit a red-shift.

**Figure 4:**
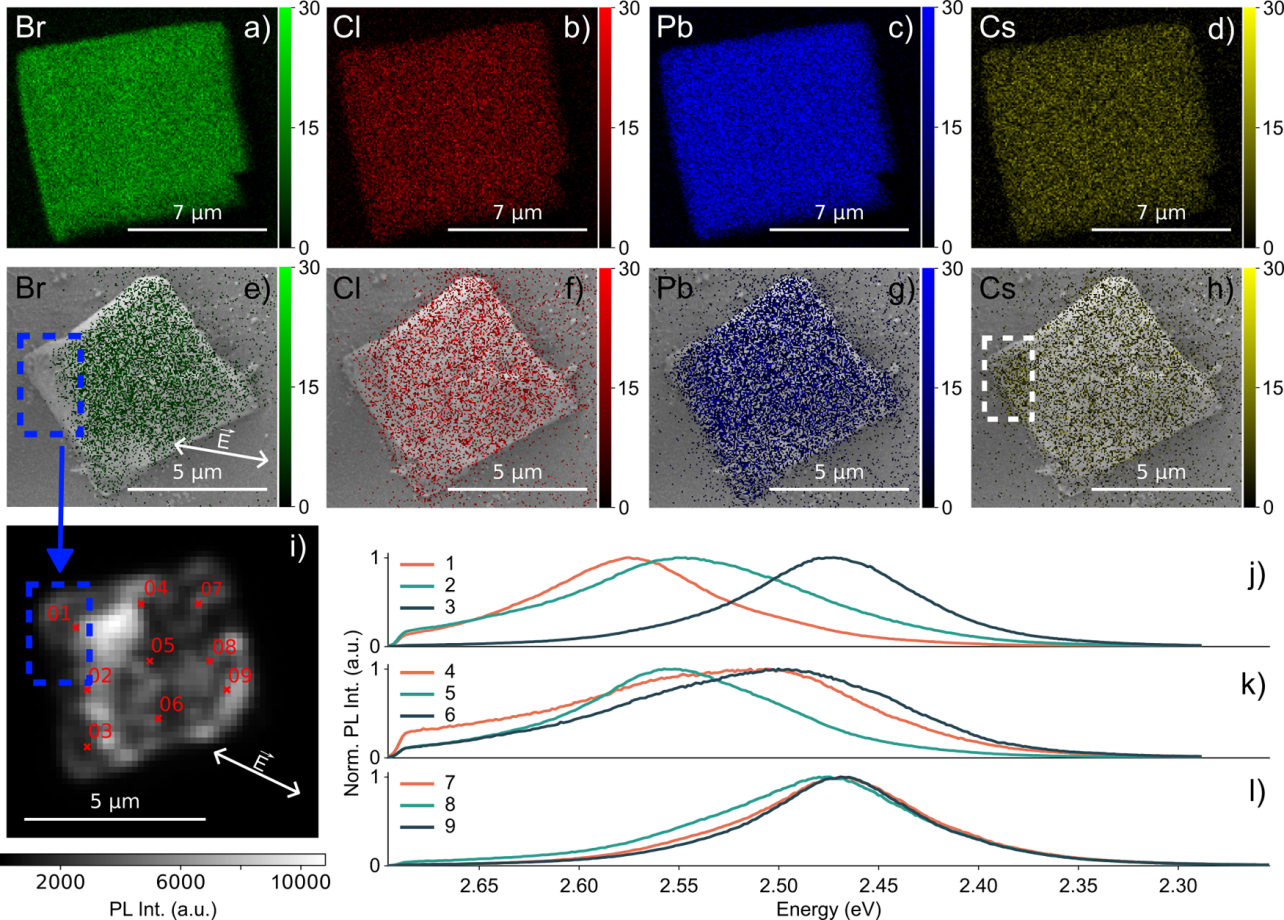
(a–d) Energy-dispersive X-ray (EDX) maps showing elemental distribution of bromide, chloride, cesium and lead in a representative CsPbBr_2.4_Cl_0.6_ SL before the exposure to an electric field. (e–h) Corresponding EDX maps of a CsPbBr_2.4_Cl_0.6_ SL inside a microcapacitor with a plate-to-plate spacing of 12 μm exposed to an electric field caused by the application of a 200 V bias. The corresponding unit is the photon intensity. (i) PL intensity image of the CsPbBr_2.4_Cl_0.6_ SL from (e–h) after electric field application. (j– l) PL spectra at the points 1–9 marked in (i). White arrows indicate the direction parallel to the electric field.

We investigated the potential sublimation of chloride and bromide ions under the influence of an applied electric field on SLs grown on silicon wafers. To this end, we employ in situ mass spectrometry on a sample subjected to an electric field and compare it to a reference measurement of a sample under identical conditions but without the application of a field. A detailed description of the experimental setup and procedure, as well as the corresponding spectra, are provided in the SI (Figure S3).

No signals corresponding to chloride or bromide species were detected in the mass spectrum of the unbiased reference sample (Figure S3a–c). In contrast, the sample exposed to an electric field exhibited peaks at 79 and 81 u consistent with the natural isotopic distribution of bromine, indicating bromide sublimation. No peaks attributable to chloride sublimation were observed in either sample.

The PL spectra in Figure 2b and the EDX data in Figure 4a–d of the SLs at zero bias confirm the mixed halide composition of the constituting LHP NCs in terms of the proposed CsPbBr_2.4_Cl_0.6_ formula. While pure CsPbBr_3_ and CsPbCl_3_ SLs are reported to exhibit a wide peak PL range around 2.4 eV^18,25,35,36^ and 3.0 eV,^18,37,38^ respectively, the PL spectra of the SLs in Figures 2 and 3 show closer relation to pure CsPbBr_3_ SL, indicating a higher bromine content. The uniform distribution of all elements and the spatially invariant PL imply that the investigated SLs are initially highly homogeneous in terms of their elemental distribution. We suggest that the transition from such an initially homogeneous state to the strongly spatially dependent PL (Figure 2c), and elemental composition (Figure 4e–h) is the result of halide diffusion. This phenomenon is not only well-documented for LHP thin films but also corroborated by the correlation of PL peak wavelengths with the varying bromide content in Figure 4e and j. We stress that for LHP NC SLs, halide diffusion over macroscopic distances in the solid state – even within a strong electric field - is not immediately obvious, since all NCs are separated by a shell of organic ligands. A potential alternative to such interparticle halide diffusion is local sublimation of halides, a process that has been observed for lead iodide perovskite NCs under prolonged optical excitation.^32–34^ Our mass spectrometry results support the possibility of bromide sublimation under an applied electric field, as evidenced by clear peaks at 79 and 81 u (Figure S3), consistent with the natural isotope pattern of bromine. These findings align with our hypothesis that electric fields may induce halide loss in LHP NC SLs. Notably, no signals indicative of chloride sublimation or related fragmentation products were detected, suggesting a potentially higher stability of chloride within the lattice or a different mechanism governing its retention. This selective bromide sublimation may contribute to the observed compositional and optical changes. We would expect that sublimation of halides should lead to a shrinkage of the overall superlattice. In contrast, the partial expansion of the superlattice visible in Figure 2a–c is more in line with the mere relocation of halides within the superlattice at the expense of the formation of defects and lattice distortion. This would also explain the occasional short-circuiting we observed, implying that field-induced expansion brought the SL into contact with both capacitor-plates. Thus, we hold interparticle halide diffusion to be the dominant mechanism behind the compositional changes that we observe under the influence of an electric field. However, as the spectroscopic data are highly surface-sensitive due to the strong absorption coefficient of the NCs, it remains uncertain to what extent halide segregation occurs throughout the SL volume. Predominantly surface-localized halide redistribution is plausible, as the electric field is likely screened with increasing depth. The SL might slowly re-equilibrate its surface halide composition with that of the interior. This process could contribute to the observed gradual shift back toward the initial emission energy despite the continued presence of the applied bias (Figure 2e, Figure 3e).

We attribute the spatial variations of the PL peak wavelengths in Figure 2d and Figure 4i to the spatially dependent strength of the applied electric field. To substantiate this, we perform finite-element modeling using the multiphysics simulation software COMSOL (for further details, see Section 7 -SI). We model a CsPbBr_2.4_Cl_0.6_ SL as a dielectric cube embedded in a parallel-plate capacitor geometry, as illustrated in Figure 5a. The simulation reveals that the introduction of a dielectric object redistributes the initially uniform electric field, as shown in Figure 5b. Upon application of an electric field, the dielectric becomes polarized due to the accumulation of bound surface charges on its facet perpendicular to the field direction. This polarization leads to the formation of an internal electric field that counteracts the applied external field resulting in a net field attenuation within these regions. Notably, the highest electric field strengths are found at the corners of the dielectric cube, where edge enhancement effects dominate. These effects cause electric field lines to converge and concentrate at geometrically sharp features, particularly along the edges that are parallel to the direction of the applied electric field. As a result, these regions experience significant local field enhancement compared to the cube center or facets perpendicular to the field. To further evaluate the internal field distribution, we analyzed electric field profiles along planar slices through the dielectric cube in XY, XZ and YZ orientations, as presented in Figure 5c–e. The results indicate that regions near the edges facing the capacitor plates (Figure 5c, d) experience a relatively attenuated electric field, consistent with surface polarization effects. However, as shown in Figure 5e regions along edges parallel to the applied field exhibit pronounced electric field enhancement relative to the center of the cube.

**Figure 5:**
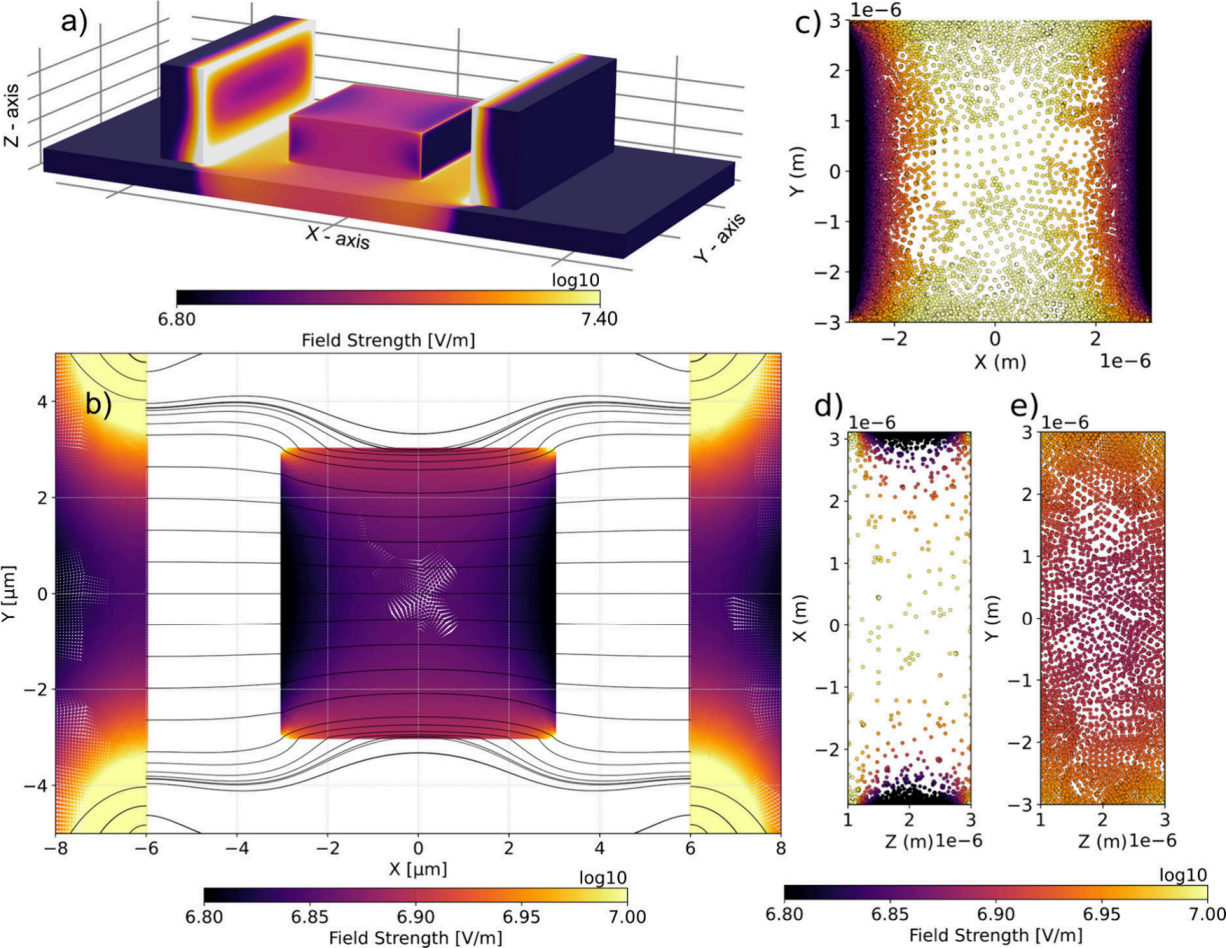
(a) Overview of the finite-element modeling of a dielectric cube between a microcapacitor. (b) Resulting electric field distribution. (c–e) Planar slices in the XY (c), XZ (d), and YZ (e) planes. The field strength color bar below (e) relates to the planes (c–e).

Our simulation implies that halide diffusion should occur around the edges of the superlattice that are parallel to the applied electric field, where the local field strength is the highest. Therefore, electric-field-induced ion diffusion is in line with the observed red-shift at these positions due to bromide ion accumulation. Likewise, the blue-shift in regions with low field strengths is likely the result of bromide depletion and a higher relative chloride concentration. Under these conditions, the diffusion of chlorides would be strongly hindered due to the positively charged vacancies left behind by the bromides with substantial screening of the electric field.

We conclude with the observation that the apparent irreversibility (Figure 2c) and gradual evolution of the PL peak (Figure 3f) cannot be explained with a potential Stark effect that one may expect for semiconductors within a strong electric field.

We investigated the impact of strong external electric fields on the structural and optical properties of self-assembled CsPbBr_2.4_Cl_0.6_ nanocrystal superlattices. Our results reveal pronounced, spatially dependent compositional and morphological changes induced by an electric field. PL shifts, supported by EDX, suggest a halide ion redistribution across the SL. Finite-element modeling suggests a nonuniform electric field distribution with enhanced electric field strength at edges parallel to the applied bias. This spatial variation drives bromide accumulation in high-local field strength regions resulting in a red-shift, as well as bromide depletion with blue-shifts in low-local field strength regions. Mass spectrometry further suggests bromide sublimation as a complementary mechanism to interparticle diffusion. The irreversible spatial PL shifts and morphological distortions point to field-induced halide mobility and structural reconfiguration as the dominant cause of the optical changes under bias, and not a Stark effect.

Our work suggests that sufficiently strong electric fields can induce inter-NC ion migration even in the presence of insulating organic ligands. In our study, this process may be facilitated by the incomplete ligand surface coverage resulting from ligand-stripping during the applied two-layer phase diffusion assembly using acetonitrile antisolvent.^22,39^ This finding is relevant for quantum dot-based perovskite solar cells, where efficient charge transport typically requires partial ligand removal or exchange with shorter, less insulating ligands.^5,41,42^ In thin absorber layers, the electric field can reach tens to a hundred kV/cm (e.g., a 1.2 V voltage across a 200 nm thick absorber layer corresponds to 60 kV/cm), comparable to the electric field strengths applied in our experiments. As shown here, interparticle ion migration may occur under such operating conditions and should be considered when evaluating the stability and long-term behavior of quantum dot-based perovskite devices.

## Supplementary Material



## Notes

Materials and methods used in the preparation and analysis of all samples. Details on the experimental techniques: Confocal laser scanning microscopy, mass spectrometry, EDX, X-ray diffraction, and on finite-element modeling. Additional information on spectral dynamics and structural changes of SLs under biasing of the microplate capacitors (PDF)
