# Supplementary material for: Interparticle Ion Migration in Cesium Lead Mixed-Halide Perovskite Nanocrystal Superlattices
Source: Nano Lett. 2026 Mar 27;26(13):4462–70. doi: 10.1021/acs.nanolett.6c00452 (PMC13067379; doi:10.1021/acs.nanolett.6c00452)
Supplement: Supplementary file 1 [file nl6c00452_si_001.pdf]

Supporting Information

Interparticle Ion Migration in Cesium Lead Mixed-Halide Perovskite Nanocrystal Superlattices

Ata Bozkurt,[1‖] Jonas L. Hiller,[1‖] Robert Thalwitzer,[1] Mario Martin,[1] Ivan Musil,[2,3] Elke Nadler,[1] Ross Ewan Carter,[2] Martin Eberle,[1] Jonas Haas,[2,3] Ivan A. Zaluzhnyy,[2] Jannik C. Meyer,[2,3] Frank Schreiber,[2] Marcus Scheele[1]*

[1] Institute of Physical and Theoretical Chemistry, University of Tübingen, Auf der Morgenstelle 18, 72076 Tübingen, Germany

[2] Institute of Applied Physics, University of Tübingen, Auf der Morgenstelle 10, 72076, Tübingen, Germany

[3] NMI Natural and Medical Sciences Institute at the University of Tübingen, Markwiesenstraße 55, 72770 Reutlingen, Germany

‖ A.B. and J.L.H contributed equally to this work,

*Corresponding author: Marcus Scheele – marcus.scheele@uni-tuebingen.de

1. Materials and Methods:

1.1 Lithography

The electrode structure was defined by optical lithography. Glass substrates (MENZEL Cover Slips) were cleaned according to Schedel et al.[1] and functionalized with hexamethyldisilazane (HMDS). The samples were spin-coated (3000 rpm for 30 s) with ma-P 1215 (micro resist technology) photoresist. Optical lithography (dose: 300 mJ/cm^{-2}) was performed with a µMLA maskless aligner (Heidelberg Instruments) using a predefined self-drawn pattern. After 25 - 30 s of development in maD-331/S (micro resist technology), thin layers of metal were deposited (2.5 nm Ti followed by between 15 nm – 20 nm Au) in a PLS570 vacuum evaporation system (PFEIFFER). The final lift-off was performed in acetone.

1.2 Deposition of microplate capacitors and energy-dispersive X-ray spectroscopy

After the lithographic process of creating the gold structures, platinum-based plate capacitors were produced using an Auriga 40 field-emission scanning electron microscope (Zeiss) equipped with an Orsay Physics Canyon focused ion beam and a organoplatinum precursor ($C_3H_6PtCpCH_3$) in a UniGIS gas injection system (Zeiss). Elemental composition was analyzed using the integrated Ultim Max 100 (Oxford Instruments) EDX detector mounted on the Auriga 40 (Zeiss) and operated with the Aztec software (Oxford Instruments). EDX measurements were conducted at an acceleration voltage of 10 kV, a working distance of 5.5 mm, an aperture size of 60 µm, high-current mode, and a specimen tilt of 30°. The elements Cs, Pb, Cl and Br were included in the analysis. Elemental maps shown in **Figure 4a-h** were acquired at an acceleration voltage of 10 kV. Secondary electron images corresponding to **Figure 4e-h** were recorded at 5 kV to minimize charging effects observed at higher voltages. The True-Map acquisition mode was employed to suppress bremsstrahlung related artifacts. Quantitative analysis of the SL composition was performed using the ESPRIT 1.9 software (Bruker) with a P/B-ZAF correction procedure to determine atomic concentrations. The analytical uncertainty of the measurements was below 3% (3σ confidence level).

1.3 Mechanical Manipulation of SLs and HR-SEM Imaging

Mechanical manipulation of the $CsPbBr_{2.4}Cl_{0.6}$ SLs was performed inside a LEO Gemini 1550 VP scanning electron microscope (Zeiss) using MM3A-EM SEM-compatible micromanipulators (Kleindiek Nanotechnik) equipped with MGS2-EM microgrippers (Kleindiek Nanotechnik). Working distances were selected to minimize charging effects while still allowing sufficient space for the insertion of micromanipulators. Acceleration voltages of 2.5 kV, an aperture of 30 µm, high current mode and a tilt of 0° were used for SL handling on both glass and silicon substrates

as well as for imaging. High-resolution SEM imaging was performed using a SU8030 (Hitachi) SEM at acceleration voltages of 1.0 and 5.0 kV. Images were acquired at magnifications of 5000x and 100000x, with a working distance of 6.5 mm and an emission current of 9.4 µA.

1.4 Optical measurements and electric field generation

Optical experiments were performed using a custom-built, inverted confocal laser stage scanning microscope. A 405 nm laser diode (LDH P-C-405, PicoQuant GmbH) was employed as the excitation source, supplying picosecond pulses with adjustable repetition rates from 10 to 80 MHz. Focusing of the excitation laser and collection of scattered and emitted light was achieved with an infinity corrected 100x/0.90 numerical aperture air objective (Carl Zeiss AG, Germany). The laser line was removed by a 458 nm long-pass filter (RazorEdge LP Edge Filter 458 RU). Time-resolved photoluminescence data were acquired by a single photon avalanche diode (PDM series, Micro Photon Devices) with an active area of 100 µm coupled with time-correlated single photon counting electronics (HydraHarp 400, PicoQuant GmbH). Scanning of the piezo stage was controlled by the HydraLabX1 controller and the HydraScan software (HydraSpex UG). Spectra were recorded by an Andor DU4A01-BVF camera attached to an Andor SR-303i-B spectrometer, using either a 300 g/mm or a 1200 g/mm grating. The temperature of the camera was set to - 60 °C. Spectra acquisition was controlled by the Andor Solis software package (Oxford Instruments). For the spectral mapping, the spectrometer was controlled by the HydraLabX1. Optical measurements were performed under ambient conditions. Electric fields were generated by applying a bias to the microcapacitors using a Keithley 2634B SourceMeter (Tektronix). The field orientation is defined by the capacitor geometry, however, the absolute direction of the electric field vector for a given bias polarity could not be unambiguously assigned.

1.5 Synthesis of $CsPbBr_{2.4}Cl_{0.6}$ NCs and assembly of NCs into SCs

Chemicals

1-Octadecene (ODE), technical grade, 90%, Sigma-Aldrich; oleic acid (OA), 97%, Acros Organics; oleylamine (OLA), 80–90%, Acros Organics; cesium carbonate (Cs_2CO_3), 99.99% (trace metal basis), Acros Organics; lead(II) acetate trihydrate (PbOAc), 99.99% (trace metal basis), Sigma-Aldrich; *n*-hexane, 97% extra dry over molecular sieve, AcroSeal, Acros; and acetonitrile, 99.9% extra dry over molecular sieve, AcroSeal, Acros. All chemicals were used as purchased.

$CsPbBr_{2.4}Cl_{0.6}$ NCs

8 nm $CsPbBr_{2.4}Cl_{0.6}$ NCs were synthesized by hot injection according to a modified literature method by Dutta et al.[2] First, 227 mg (0.6 mmol) of PbOAc and 97 mg (0.3 mmol) of Cs_2CO_3 were added to a 50 mL three-neck flask containing 3 mL of OA and 30 mL of ODE. The flask was degassed under vacuum at 120 °C for 2 hours, after which the temperature was increased to 240 °C. 3 mL of an OLA-HX precursor solution (1.1 M, mixture of 2 mL OLA-HBr and 1 mL OLA-HCl) was injected, which are prepared by dissolving 0.64 mL HBr and 0.5 mL HCl respectively in 5 mL OLA. The NCs were centrifuged at 10000 rpm for 1 min. The supernatant was then discarded, and the crude precipitate was centrifuged at 10000 rpm for 10 min. Any traces of the supernatant were removed after which the precipitate was dissolved in 3 mL hexane. The NC-hexane dispersions were filtered through a 0.2 µm PTFE syringe before storage in a glovebox.

SC growth via two-layer diffusion technique

To grow the SLs, silicon wafers measuring 10 × 10 mm² were used as substrates and processed in a nitrogen atmosphere. Each wafer was placed inside a 10 mm diameter test tube, which was then inserted into a 50 mL centrifuge tube. A biphasic solvent system was prepared by adding 600 µL of acetonitrile on top of 600 µL of an approximately 2 µM NC solution in hexane. The tubes were sealed, wrapped in aluminium foil and left at room temperature for five days to allow crystallization to occur. After this period, residual solvents were removed, and the samples were left to dry for two hours. Final inspection under an optical microscope confirmed the presence of microscopic single crystals on the substrate surface.

2. Confocal microscopy

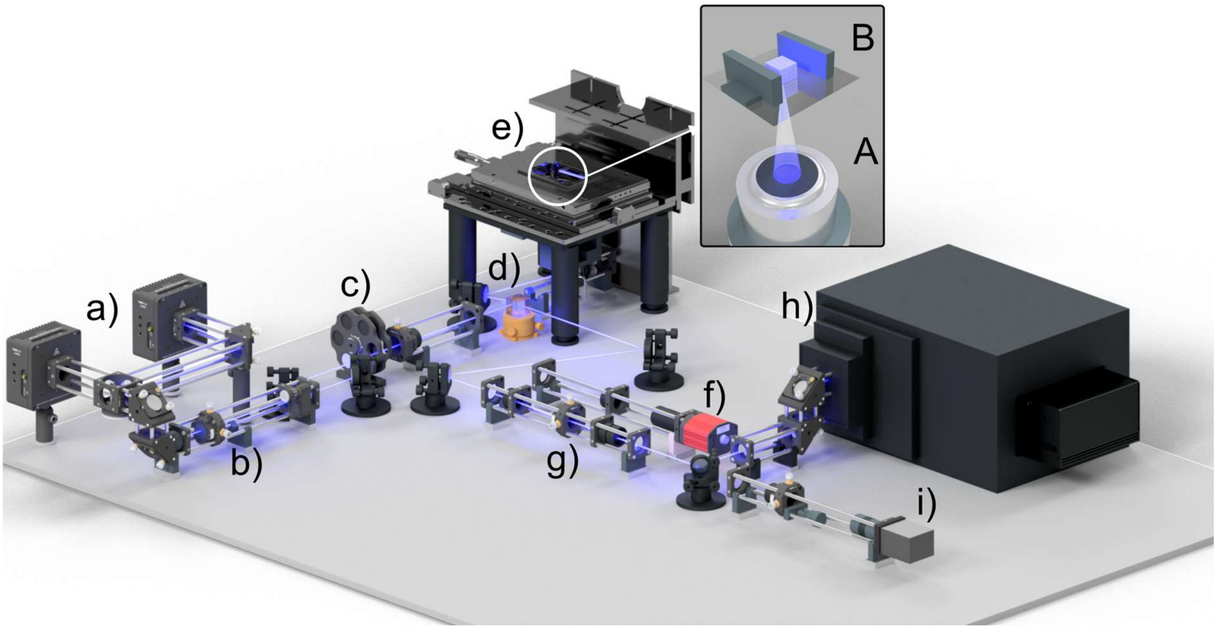

Figure S1: Overview of the confocal microscope setup, depicting a) the laser diode, b) the refractive telescope, c) the optical density filter wheel, d) the 50/50 beam splitter, e) the sample stage, f) the imaging camera, g) a second refractive telescope equipped with a long-pass filter, h) the spectrometer, and i) the avalanche photodiode.

The confocal microscopy setup is schematically depicted in **Figure S1**. The naturally divergent beam from the employed laser diode (**Figure S1a**) is collimated by directing it through a refractive telescope (**Figure S1b**) with a pinhole at the shared focal point. The excitation intensity can be tuned using an optical density filter wheel (**Figure S1c**). The collimated excitation beam is directed through a 50/50 cube beam splitter (**Figure S1d**) and is focused by an objective lens (A) on the sample (B) mounted on a piezo-driven stage (**Figure S1e**).

Scattered and emitted light from within the focal volume are collected by the same objective and are again directed through the 50/50 beam splitter cube. The positioning of the beamsplitter cube in the optical setup ensures that all light in the detection path (f-i) either originates from the sample in the focus (photoluminescence) or has interacted with it (scattering). Using a flip-mirror, the light in the detection path is either directed to an imaging camera (**Figure S1f**) or to a second refractive telescope (**Figure S1g**) with a pinhole at the shared focal point. For photoluminescence measurements, a suitable long-pass filter is installed at the end of the second telescope. Another flip-mirror located behind the telescope directs the beam either to a spectrometer (**Figure S1h**) or to a single-photon avalanche photodiode (**Figure S1i**).

For the spectral maps presented in this work, the SL sample is scanned through the diffraction-limited focal volume, and photoluminescence spectra are recorded by the spectrometer at each position of the scan. From the recorded spectra, maps of properties of the spectra, e.g. the peak position, are generated. To study the influence of the electric fields, the plates of the microcapacitors were connected to a voltage source. Spectral maps were recorded before and after the application of a bias. The piezo stage was also used to move a specific position on the SL sample into focus and continuously record spectra from that position.

3. Mass spectrometry

3.1 Measurement without bias

Mass spectrometry was used to analyze gases released from the SLs while they are placed inside a vacuum. For this purpose, $CsPbBr_{2.4}Cl_{0.6}$ SLs were assembled on a 10 × 10 mm² silicon wafer with a silicon oxide layer thickness of 750 nm. The sample was inserted into a vacuum chamber. Vacuum was maintained by the combination of a turbomolecular pump and ion pump, reaching pressures on the order of 10^{-5} Pa. Close to the sample, a *XT300M Residual Gas Analyzer* (*Extorr)* was mounted, which utilizes electron impact ionization, and a quadrupole analyzer coupled with an electron multiplier.

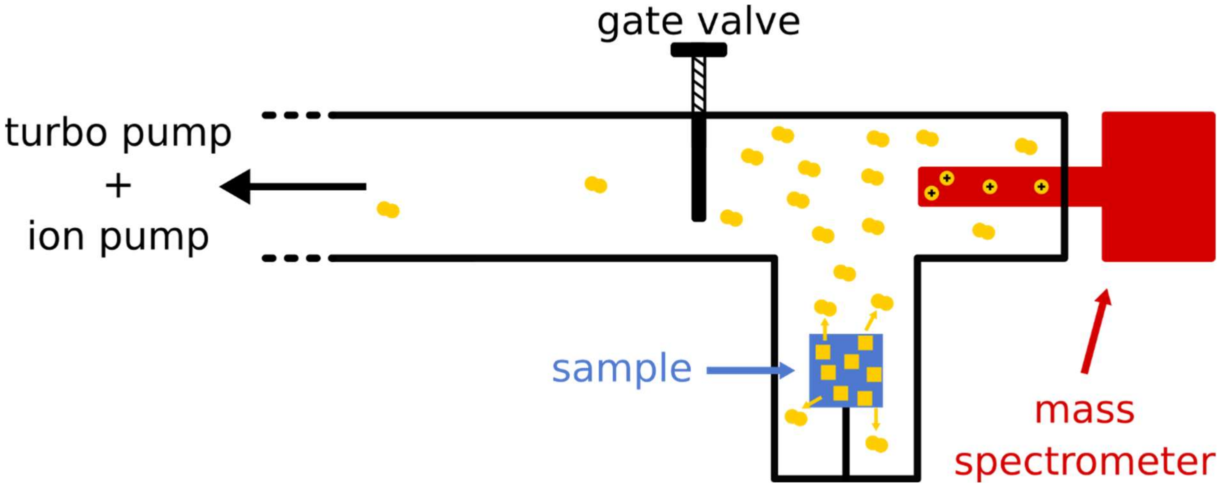

Figure S2: Schematic drawing of the setup used for measuring gaseous species leaving the sample. Any gas coming from the sample is partially trapped by the gate valve and can therefore reach the mass spectrometer more efficiently.

Figure S2 shows a schematic scheme of the setup. Before each measurement, a gate valve, separating mass spectrometer and sample from the pumps, was slowly closed to hamper the diffusion of gas to the pumps, but left open wide enough to still allow for a vacuum in the upper 10^{-5} Pa range. By partly closing the gate valve, the probability for any gases from the sample reaching the mass spectrometer is increased.

3.2 Measurement with bias

The same wafer preparation was done as described in Section 3.1. A second wafer (same type), with 18 × 18 mm², was placed on top of the SL layer as a counter electrode. Both wafers were electrically contacted on the backside by grinding away the oxide layer, sputtering gold onto it and connecting them with copper wires via electrically conductive silver adhesive. This way, a capacitor was formed between the conducting (doped) bulk of the silicon chips and the electrical separation of the electrodes consisting of the two 750 nm oxide layers on each side of the $CsPbBr_{2.4}Cl_{0.6}$ SLs. The sample was inserted into a vacuum chamber and connected to the outside by an electric feed-through. Vacuum was maintained as described in the measurements without bias. Both electrodes were connected to a source measure unit and up to 210 V bias was applied and recorded by a multimeter to monitor if any current is flowing through the capacitor (e.g. because of a short circuit).

3.3 Further results and discussion

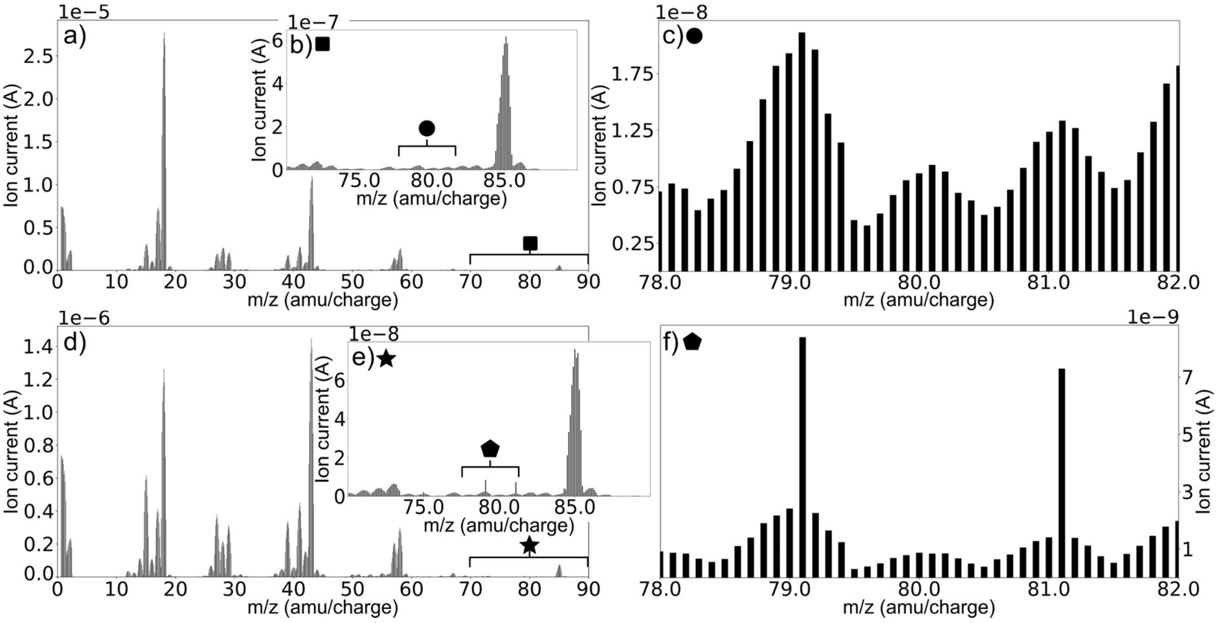

Figure S3: a) Mass spectra of a wafer containing $CsPbBr_{2.4}Cl_{0.6}$ SLs without applied bias. The unit amu (= u) refers to the atomic mass unit. b) Magnified view of the 70 to 90 u region. c) Detailed spectrum highlighting bromide-related signals. d) Corresponding spectra from another wafer measured under applied bias. e) Magnified view of the 70 to 90 u region. f) Detailed bromide signals.

Figure S3 presents the mass spectra obtained from the analysis of $CsPbBr_{2.4}Cl_{0.6}$, as described in Section 3.1. **Figures S3a-c** show spectra from a wafer containing SLs without applied bias, while **Figures S3d-f** correspond to measurements with applied bias. Prominent signals are observed at 79.1 u and 81.1 u, exhibiting an isotope pattern with a m+2 spacing and an intensity ratio of approximately 1:0.95, consistent with the presence of bromide. In contrast, no significant signals attributable to chloride or its fragments (typically expected to exhibit m+2 pattern with a 3:1 intensity ratio) are detected. In **Figures S3a** and **S3d**, signals in the 0-10 u range likely correspond to residual gases from the vacuum chamber. Additional signals and their tentative assignments are summarized in **Table S1**.

Table S1: List of fragments assigned to the detected masses in Figure S3 and the assignment to the source of the fragmentation.

Source Chemical	Fragment	m/z
Hexane/1-Octadecene	C_1H_3 Alkyl/Alkenyl	15.0
Hexane/1-Octadecene	C_1H_1 Alkyl/Alkenyl	13.0
Hexane/1-Octadecene	C_2H_5 Alkyl/Alkenyl	29.0
Hexane/1-Octadecene	C_2H_3 Alkyl/Alkenyl	27.0
Hexane/1-Octadecene	C_3H_7 Alkyl/Alkenyl	43.1
Hexane/1-Octadecene	C_3H_5 Alkyl/Alkenyl	41.0
Hexane/1-Octadecene	C_4H_7 Alkyl/Alkenyl	55.1
Hexane/1-Octadecene	C_4H_9 Alkyl/Alkenyl	57.1
Hexane/1-Octadecene	C_5H_9 Alkyl/Alkenyl	69.1
Hexane/1-Octadecene	C_5H_{11} Alkyl/Alkenyl	71.1
Hexane/1-Octadecene	C_6H_{11} Alkyl/Alkenyl	83.1
Hexane/1-Octadecene	C_6H_{13} Alkyl/Alkenyl	85.1
1-Octadecene	C_7H_{15} Alkyl/Alkenyl	99.1

Source Chemical	Fragment	m/z
Acetonitrile	CH_2N^+	28.0
Acetonitrile	CN^+	26.0
Oleylamine	$CH_2CH_2NH_2^+$	44.1
Oleylamine	$C_3H_8N^+$	58.1
Oleylamine	$(CH_2NH_2)^+$	30.0
Oleic acid	Carboxyl group	45.0
$CsPbBr_{2.4}Cl_{0.6}$	Br^+	79.1 / 81.1
Hexane	Parent Ion	86.1
Acetonitrile	Parent Ion	41.0

There was a significant difference in the overall ion currents when comparing **Figure S3a** and **Figure S3d**, which we attribute to changes in the spectrometer's sensitivity, since the relative strengths of the signals (except for Br)

remained similar. **Figure S4** provides visual evidence supporting our hypothesis of sublimation. Following the application of an electric bias, a residual buildup was observed near the plate capacitors surrounding the superlattice structure. This residue (outlined by blue dashed lines) is presumed to consist partially of organic material, likely ejected due to the influence of the strong electric field. This interpretation is supported by the mass spectra in **Figure S3a** and **S3d**, which reveal strong organic signals.

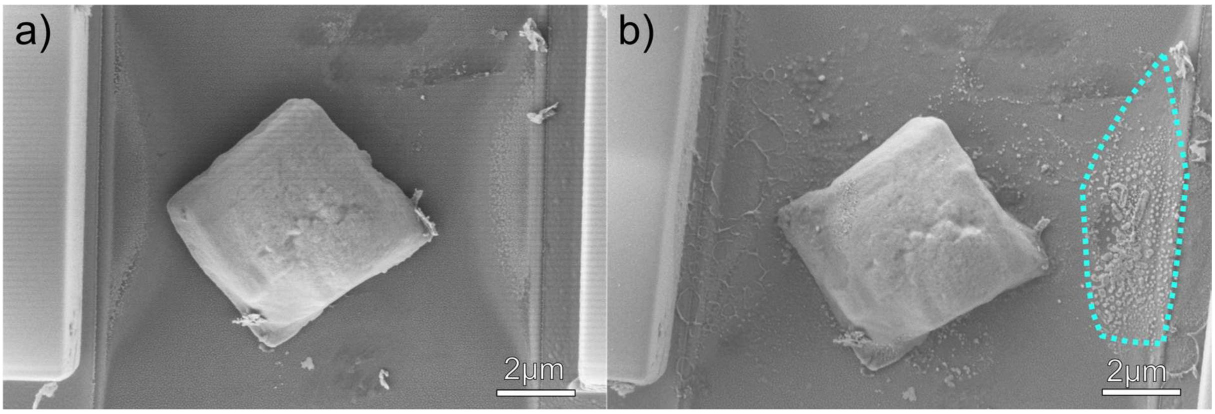

Figure S4: a) SEM images of a $CsPbBr_{2.4}Cl_{0.6}$ SL before the application of an electric field. b) $CsPbBr_{2.4}Cl_{0.6}$ SL after the application of an electric field. The newly formed residue is highlighted with blue dashed lines in b).

4. Evolution of the emission peak energy distribution recorded from a SL under biasing

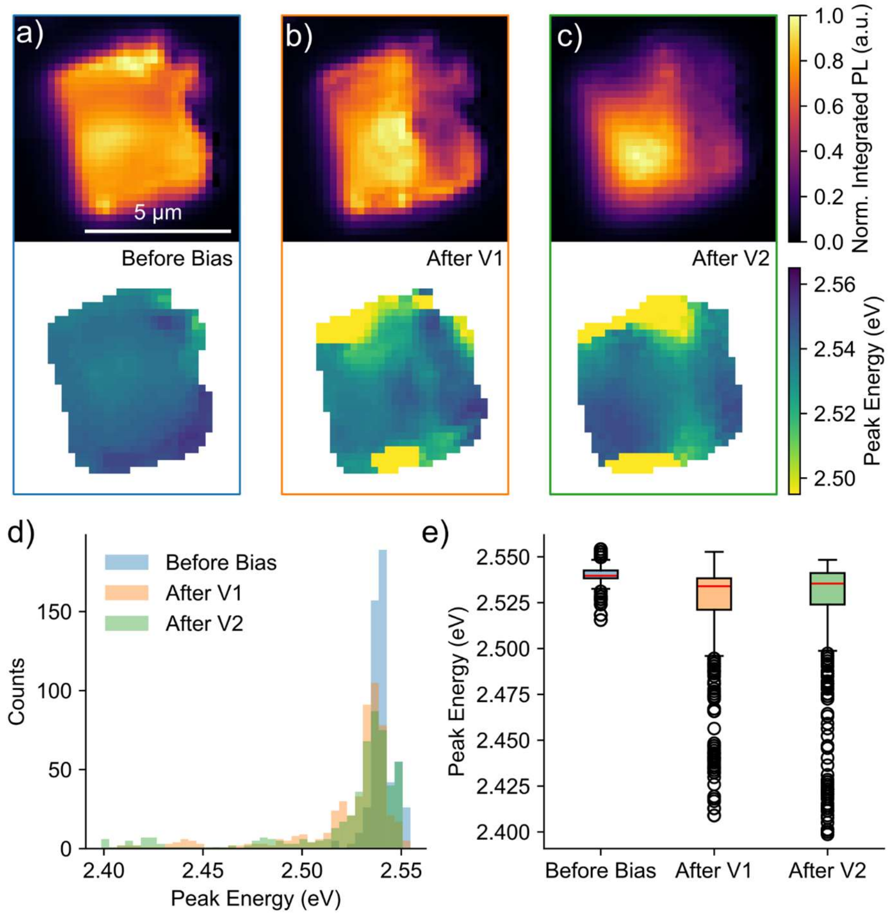

Figure S5: (a-e) Normalized integrated PL intensity maps (upper panels) and corresponding intensity-thresholded (masked) emission peak energy maps (lower panels) of the SL: a) before bias application, b) after the first application of a bias to the microplate capacitor (V1), and c) after the second application of an external bias (V2). d) Histograms of the emission peak energy distributions extracted from the masked SL region for the three conditions with a bin width of 4 meV. e) Box plots of the peak energy distributions.

We have analyzed the spatial distribution of emission peak energies recorded from the SL shown in **Figure 2** of the main text both before and after each of the two biasing steps. The results are displayed in **Figure S5** and **Table S2**.

Table S2: Median, first quartile (Q1), third quartile (Q3), interquartile range (IQR), and skewness of the peak energy distributions extracted from boxplot analysis.

	Median (eV)	Q1 (eV)	Q3 (eV)	IQR (meV)	Skewness
Before Bias	2.540	2.538	2.543	5	-0.38
After V1	2.534	2.521	2.538	17	-2.31
After V2	2.535	2.524	2.541	17	-2.22

We find that the distribution of emission peak energies of the unbiased sample is relatively narrow (IQR = 5 meV) and approximately symmetric (skewness = -0.38). After application of a voltage to the microcapacitor, this is no longer the case. The peak energy distributions broaden substantially (ΔIQR = 13 meV, approximately quadrupling), with the majority of image pixels exhibiting a red-shift leading to an asymmetric distribution (Skewness = -2.31 and -2.22 after V1 and V2, respectively). This can also be seen in the pronounced low-energy tailing of the histograms in **Figure S5d** (and in the large number of low-energy outliers of the boxplots in **Figure S5e**).

5. A closer look at the spectral dynamics observed in Figure 2e of the main text

Figure S6 displays the temporal evolution of the PL peak energy during the spectral series. Upon the application of 200 V bias to the microplate capacitor after 100 s, the emission exhibits a rapid shift to lower energies, reaching 2.46 eV within the first 5 s. This is followed by a slower, continuous red shift during the following 20 seconds with the peak energy approaching 2.42 eV (see inset). After approx. 250 s total (150 s after the biasing), the peak energy starts to gradually shift towards higher energy, plateauing at around 2.48 eV. When the 200 V bias is removed, we observe a further gradual blue shifting of the peak energy.

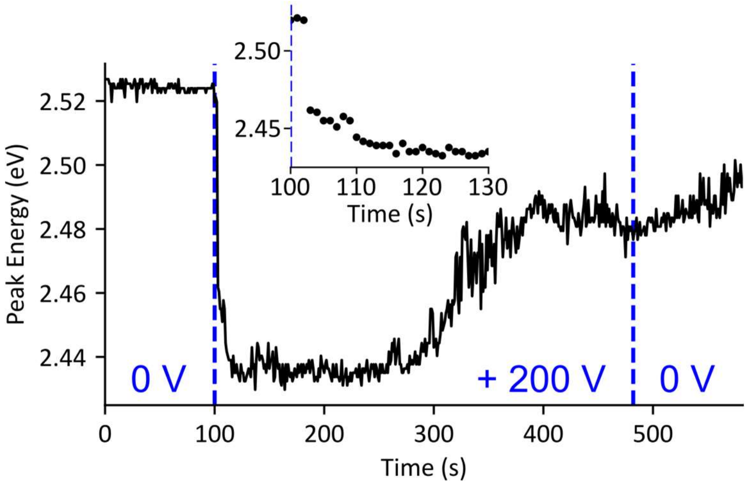

Figure S6: Temporal evolution of the PL peak energy vs time in the series spectrum recorded from the position marked by the white cross in Figure 2c and displayed in ***Figure 2e*** *of the main text. The inset displays the response in the 30 spectra/seconds following the bias application.*

Figure S7 displays the individual spectra within the spectral series under the application of the 200 V bias (100 to 482 s) split into two consecutive timeframes (99-200 s and 200-482 s). **Figure S7** shows that the evolution of the emission peak energy alone does not fully describe the spectral dynamics, which also involves changes in the PL intensity and peak shape. Periods during which the peak energy shifts appear to be accommodated by a reduction in emission intensity, whereas phases where the peak energy stabilizes coincide with a recovery of the intensity.

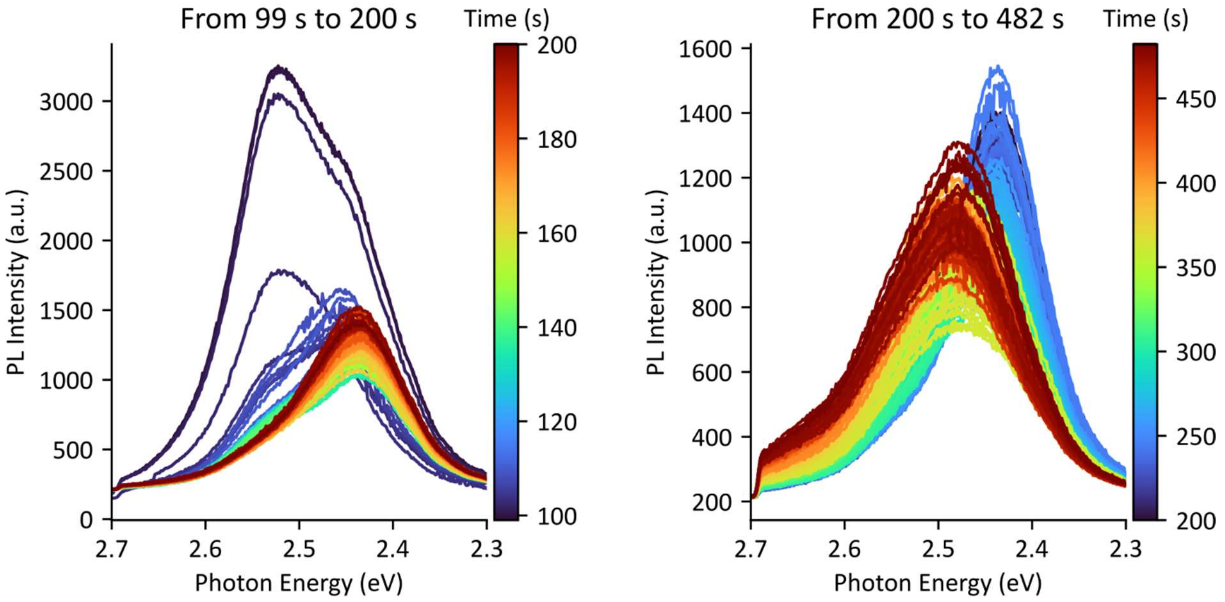

*Figure S7: Spectral series recorded from the position marked by the white cross in **Figure 2c** of the main text. The left panel displays spectra acquired between 99 s and 200 s, covering the initial response to the application of a +200 V bias to the microplate capacitor, while the right panel shows spectra from 200 s until the end of the biasing step at 482 s.*

How can we rationalize the observed dynamic behavior? First, it should be noted the spectral dynamics discussed here are specific to the position from which the spectral series was recorded. This position, marked by the white cross in **Figure 2c**, is adjacent to the edge of the crystal that exhibits red shifted emission due to the preceding biasing step (V1) performed on the sample. This prior exposure to an electric field explains the low-energy shoulder in the initial emission spectra of the series. We attribute this feature to the presence of two distinct emitting compositions in the probed focal volume (lateral focus size of approx. 250×250 nm^2, with a shallow focal depth due to the strong SL absorption): One composition is more chloride-rich (higher-energy emission) and one more bromide rich (lower-energy emission).

A full microscopic description of the observed behavior is challenging. However, we can present a plausible explanation. We suggest that the observed spectral dynamics arise from field-driven halide redistribution and the resulting partial screening of the local electric field. Upon application of the + 200 V bias, the electric field appears to preferentially drive more mobile bromide ions towards the probed region close to the crystal edge. This results in the rapid initial red shifting of the emission towards 2.46 eV. The subsequent slower red shift to around 2.42 eV over tens of seconds suggests a continued but progressively weaker redistribution process, likely limited by ionic mobility and local concentration gradients. These effects lead to a partial screening of the local electric field, to which we attribute the partial reversal of the peak energy to around 2.48 eV after extended biasing. In this regime, the emission originates from a single emissive mixed-halide composition, manifesting as a single broadened peak. This contrasts with the initially observed spectra, where two distinct emitting compositions were observed. Once the bias is removed, the observed continuous blue shift is consistent with a slow back-diffusion of halides.

6. SL destruction by short circuiting

During electrical measurements, we observed short-circuiting in samples where the SLs were tightly confined between plate capacitors, despite not being in direct contact with either electrode as seen in **Figure S8a**. Care was taken to ensure that the superlattice remained electrically isolated from both plates to prevent unintentional conduction. However, upon applying an electrical bias, a loud snapping noise and a green spark was observed, followed by a complete loss of photoluminescence signal. Subsequent inspection under an optical microscope

revealed a fragmented region of the superlattice along with substrate damage as shown in **Figure S8b**. The corresponding spectral series is depicted in **Figure S9**.

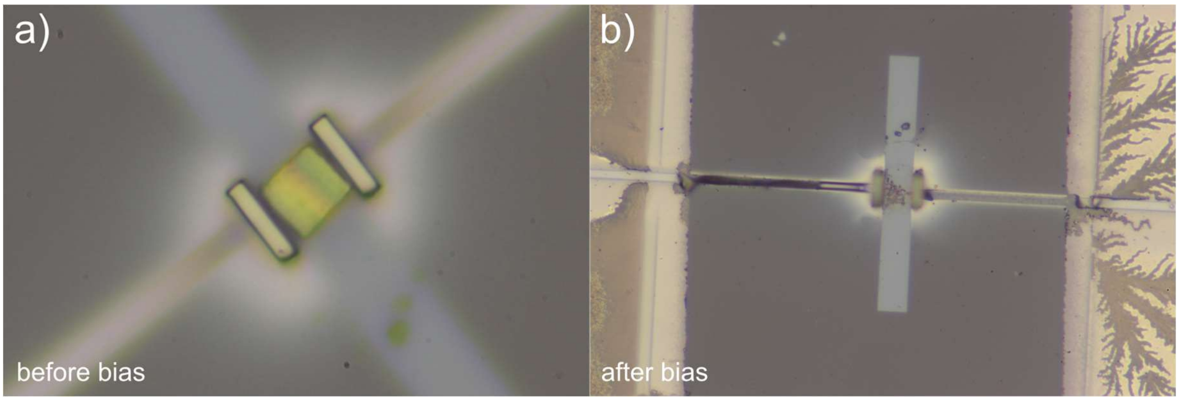

Figure S8: a) $CsPbBr_{2.4}Cl_{0.6}$ SL tightly confined between plate capacitors without direct contact to either electrode before bias application. b) $CsPbBr_{2.4}Cl_{0.6}$ SL after the application of an electric field, which caused a short circuit and destroyed the SL.

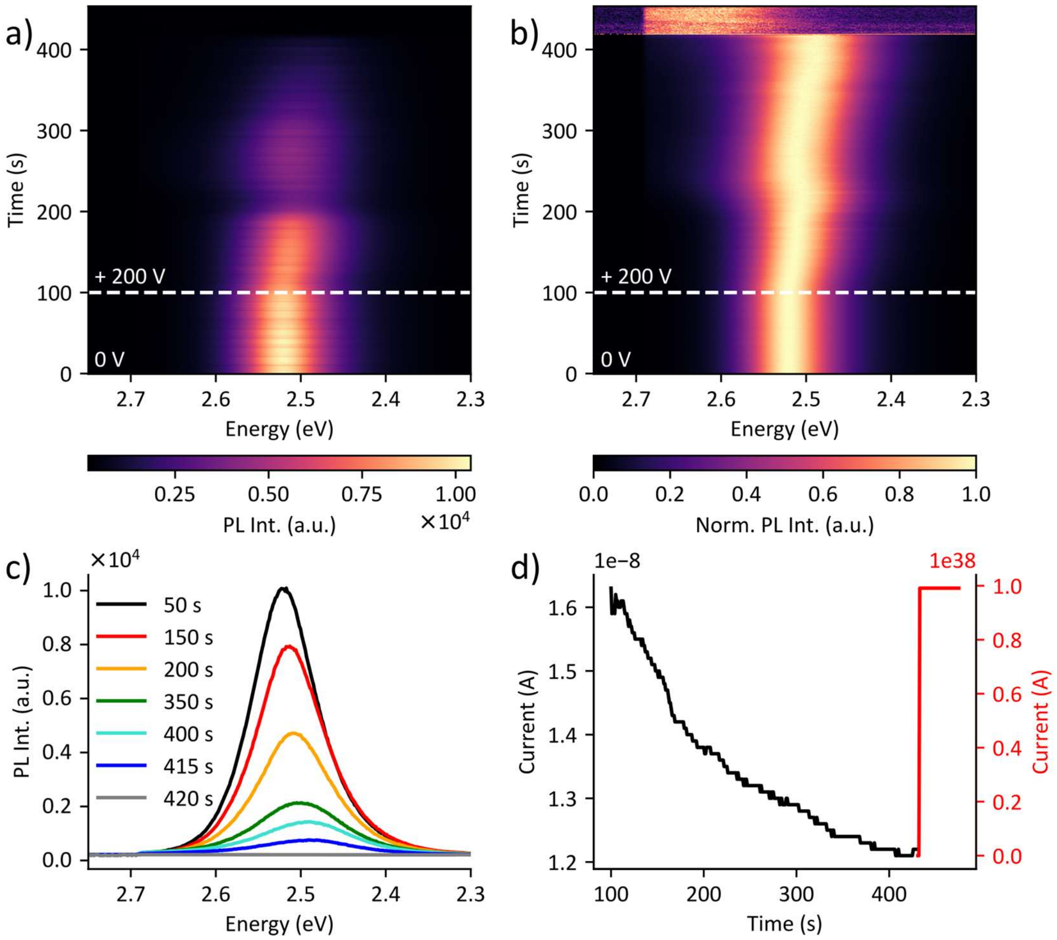

Figure S9: a) Series of PL spectra (1 second/spectra) acquired from the center of the $CsPbBr_{2.4}Cl_{0.6}$ SL shown in Figure S8. The dashed white line indicates the moment a bias of +200 V is applied to the microplate capacitor after 100 seconds. b) The normalized spectral series from panel a). c) PL spectra taken from selected timestamps of the spectral series. d) Current recorded during the spectral series starting with the application of the bias.

Figure S9a and b display a series of PL spectra recorded from the centre of the SL displayed in **Figure S8a**. After the application of a +200 V bias to the microcapacitor, an initial red-shifting and gradual decrease of PL intensity

is observed. As shown by the black line in **Figure S9d**, this is accompanied by a small and steadily decreasing flow of current between the capacitor plates. 320 seconds after the application of the bias (t = 420 s), the PL instantly and completely vanishes and current overflow between the capacitor plates is recorded (**Figure S9d** - red line), indicating the short-circuiting.

7. Further details and discussion of the finite-element modeling

We used the COMSOL Multiphysics software 5.3 for the finite-element simulation with the electric currents interface to compute electric fields in a stationary study, meaning variables are assumed to be time-independent. The simulation geometry replicated the real experimental setup. The system consisted of two pure platinum capacitor plates with dimensions of 10 µm x 2 µm x 3 µm, separated by 12 µm and placed on a glass substrate measuring 100 × 100 μm^2. The surrounding medium was air, and a cube was positioned at the centre between the plates. The cube was modelled as an insulating material with a relative permittivity of 10. An applied voltage of 200 V was used. A fine mesh was applied to the surfaces, while a very fine mesh was used at the corners and edges of both the plates and the dielectric cube to ensure accuracy in regions with high field gradients.

Although our finite-element simulation provides a clear picture of how the electric field is distributed around a dielectric cube, it remains a simplified representation of the real $CsPbBr_{2.4}Cl_{0.6}$ SL. In our model, the SL was treated as an insulating cube with a defined relative permittivity placed between two electrodes. This setup helps visualizing the field interactions but cannot fully capture the complex electronic and ionic behaviour of the actual material. In practice, LHP NCs exhibit semiconducting properties. The surrounding organic ligand shell behaves as an insulating spacer between the nanocrystals, forming an overall structure with mixed electrical character. The SL therefore behaves neither as a pure insulator nor a uniform dielectric, but as a system where local conductivity and polarization can vary significantly. Our dielectric cube model should thus be seen as an approximation of the macroscopic electrostatic response. Even with these limitations, the simulations remain valuable. It highlights regions of strong field enhancements, which aligns well with experimental observations of halide redistribution and spectral shifts.

8. Further details regarding EDX measurements

In addition to the discussion of the EDX elemental maps shown in **Figure 4**, it is important to examine the corresponding EDX spectra. **Figure S10** presents EDX spectra from the SLs in **Figure 4**. EDX Spectra from the SL subjected to an electric field (**Figure 4e-h**) are shown in **Figures S10a/c**, while the spectra for the reference SL (**Figure 4a-d**) are shown in **Figures S10b/d**. We first consider **Figures S10a/b**, where spectra were collected from measuring the entire SL and part of the underlying substrate. In the SL exposed to an electric field (**Figure S10a**), additional signals corresponding to Na and Ca are observed, consistent with the elemental composition of the glass substrate. In contrast, the SL not influenced by an electrical field (**Figure S10b**) exhibits only Si signal from the substrate, in agreement with the use of a Si substrate in this case. Both spectra show the presence of oxygen, which is attributed to surface contamination resulting from sample exposure to air. In addition, characteristic signals corresponding to the elemental composition of the SLs are detected in both cases. Similar observations apply to **Figures S10c/d**, where spectra were acquired from a small region at the centre of the SL. While no compositional differences are observed between the two samples, a pronounced difference in signal intensity is evident, where both **Figures S10a** and **c** show reduced intensity compared to **Figures S10b** and **d**.

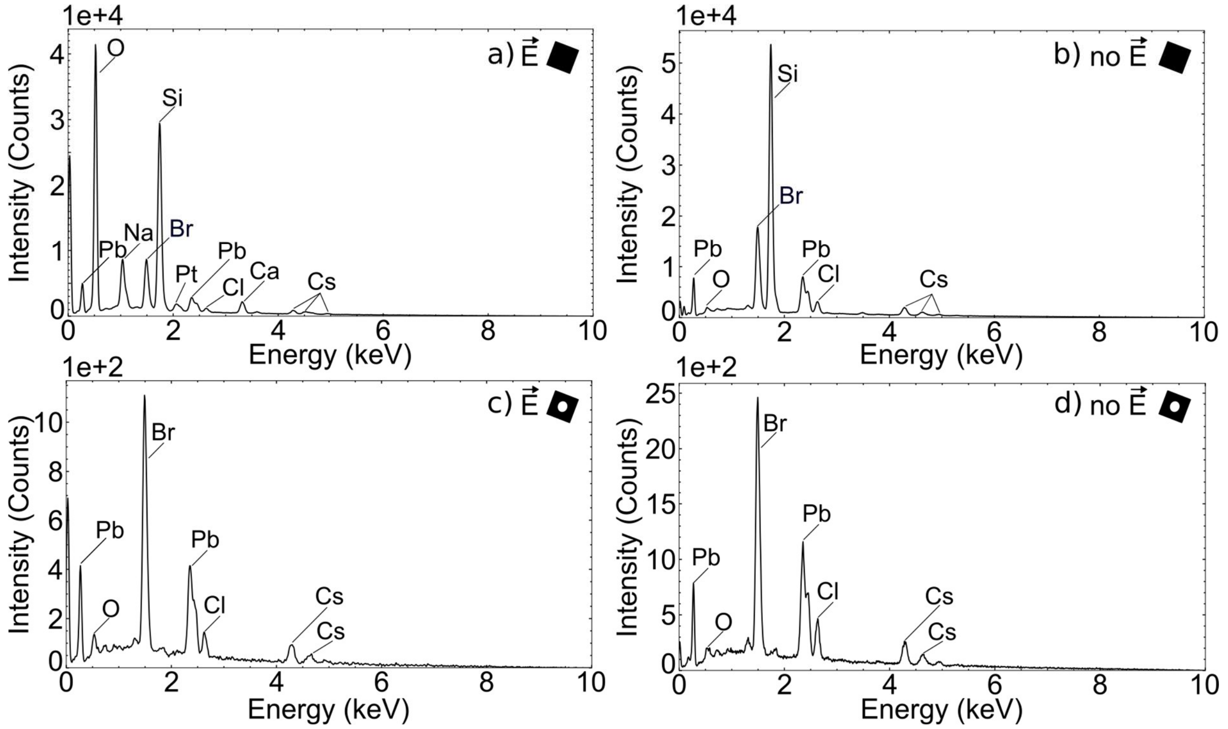

Figure S10: EDX spectra of a) the entire SL (symbolized by the black square) subjected to an electric field, b) the entire SL not influenced by an electric field, c) the central region (symbolized by the black square with a central dot) of a) and d) the central region of b).

This intensity variation originates from the different substrate materials used. An insulating and optically transparent glass substrate is required for the microplate capacitors to generate an electric field across the SLs and perform optical measurements, whereas the SLs themselves were grown on a Si substrate. Parameters for EDX measurements were therefore optimized to balance sufficient signal intensity with effective mitigation of charge build-up on the glass substrate. Prolonged electron beam exposure of the insulating glass substrate leads to the accumulation of negative charge over time. Consequently, the image would drift more with increasing charge, leading to a misalignment of individual scans, smearing out the final EDX map and adding an offset relative to the initial electron image. To mitigate this, the integration time, and therefore the amount of collected signal for the SLs exposed to an electric field on a glass substrate were limited in comparison to the measurements performed on the as-assembled SLs on conductive Si substrates, for which charging effects are negligible. This substrate-dependent difference in integration time causes the intensity variations seen in the EDX elemental maps in **Figure 4**.

9. Complementary structural analysis after the application of an electric field

We characterized two samples of $CsPbBr_{2.4}Cl_{0.6}$ SLs on Si substrates, under ambient conditions, using the X-ray laboratory diffractometer (GE-3303TT, Cu-$K_{\alpha 1}$ radiation) in θ:2θ reflection geometry between the angles $\theta =$ 5-30°. These measurements act as an average over all SLs present on the substrate.

First, two $CsPbBr_{2.4}Cl_{0.6}$ samples were fabricated on Si substrate. The native oxide layer was removed from the backside of each substrate. To ensure we have a conductive contact for the current source, a gold layer was then applied. A parallel plate capacitor configuration was then assembled by stacking the primary Si substrate onto a secondary bigger Si substrate. The two were separated by 100 µm thick Kapton spacers positioned at each corner to prevent damage to the SLs. Finally, a bias of +600 V generating an electric field with an approx. strength of 60 kV/cm was applied to the experimental sample (**Figure S11b**), while a reference was maintained at 0 V bias (**Figure S11a**)

The purpose of these measurements is four-fold: garner whether there is preferred orientation of SLs on the substrate; corroborate the likely composition of SLs based on the position of measured Bragg peaks; determine

the corresponding atomic lattice constants; and, most importantly, investigate whether the aforementioned characteristics change due to the application of +600 V.

Assuming a cubic structure, both X-ray diffraction intensity curves in **Figure S11** display only the (100) and (200) Bragg peaks. The absence of Bragg peaks that correspond to other scattering plane directions (e.g. (110) and (111)) indicates a preferred orientation for the growth of SLs, whereby the (X00) scattering planes are normal to the substrate.

Given that the expected composition of our sample is $CsPbBr_{2.4}Cl_{0.6}$, we predict to observe each Bragg peak centred between the q-values corresponding to $CsPbBr_3$ ($q_{(100)} = 1.07\ \text{Å}^{-1}$, $q_{(200)} = 2.14\ \text{Å}^{-1}$)[3] and $CsPbCl_3$ ($q_{(100)} = 1.12\ \text{Å}^{-1}$, $q_{(200)} = 2.24\ \text{Å}^{-1}$)[4], which are denoted by red and green dashed lines respectively in **Figures S11a-b**. We observe the centre of mass of both Bragg peaks in **Figures S11a-b** to be precisely in the expected range ($q_{(100)} = 1.09\ \text{Å}^{-1}$, $q_{(200)} = 2.18\ \text{Å}^{-1}$), substantiating our prior claims about composition. Taking the q-position of the first Bragg peak, we calculate the pseudocubic lattice constant $a = 2\pi/q_{(100)} = 5.76\ \text{Å}$.

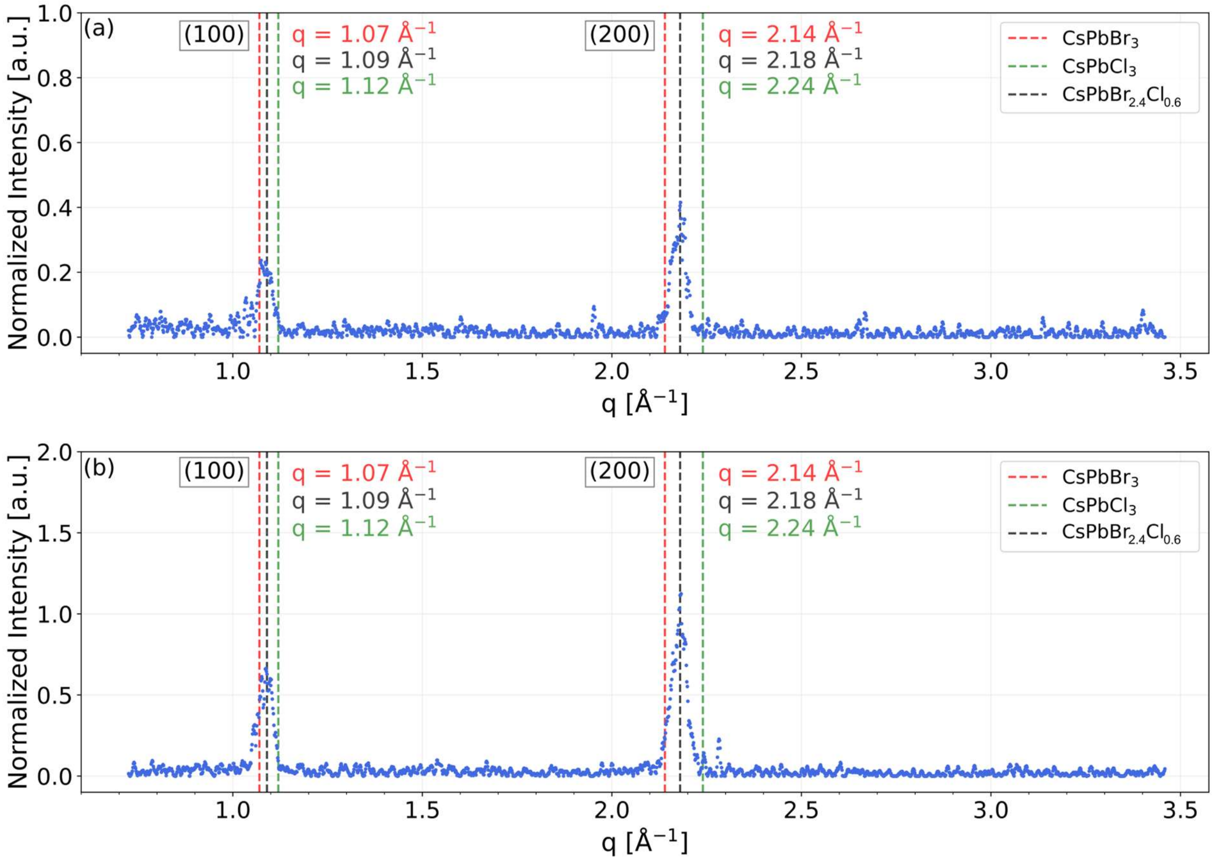

Figure S11: X-ray diffraction intensity curves of $CsPbBr_{2.4}Cl_{0.6}$ SLs a) before and b) after the application of a +600 V bias, measured in θ:2θ reflection geometry. The expected position of (100) and (200) Bragg peaks for $CsPbBr_3$ and $CsPbCl_3$ are denoted by red and green dashed lines respectively, and the centre of mass of measured Bragg peaks is indicated by black dashed lines. The experimental data was smoothed using a Savitzky-Golay filter for better presentation.

To assess the structural integrity and NC organization following an application of an external electric field, high-resolution (HR) SEM was performed (**Figure S12**). **Figure S12a** displays the representative morphology of a SL. Detailed structural analysis was conducted at the center (**Figure S12b**, magenta) and the lower central region (**Figure S12c**, cyan) of the SL. The HR SEM micrographs reveal that the long-range translational symmetry of the constituent NCs remains intact, showing regions exhibiting highly ordered packaging.

The structural periodicity of the superlattice was quantified using Fast Fourier Transformation (FFT) analysis of the HR SEM micrographs. The presence of sharp, well defined diffraction spots in the Fourier spectra (**Figures S12b/c**) confirms the retention of long-range translational symmetry following the procedure. The calculated reciprocal lattice vectors of 0.081 nm^{-1} and 0.079 nm^{-1} corresponding to a interparticle spacing of approximately 12.34 nm and 12.66 nm, indicating a uniform and highly ordered NC assembly. Significant charging effects due to the insulating nature of the glass substrate complicate the investigation of NCs at the very edges of the SL.

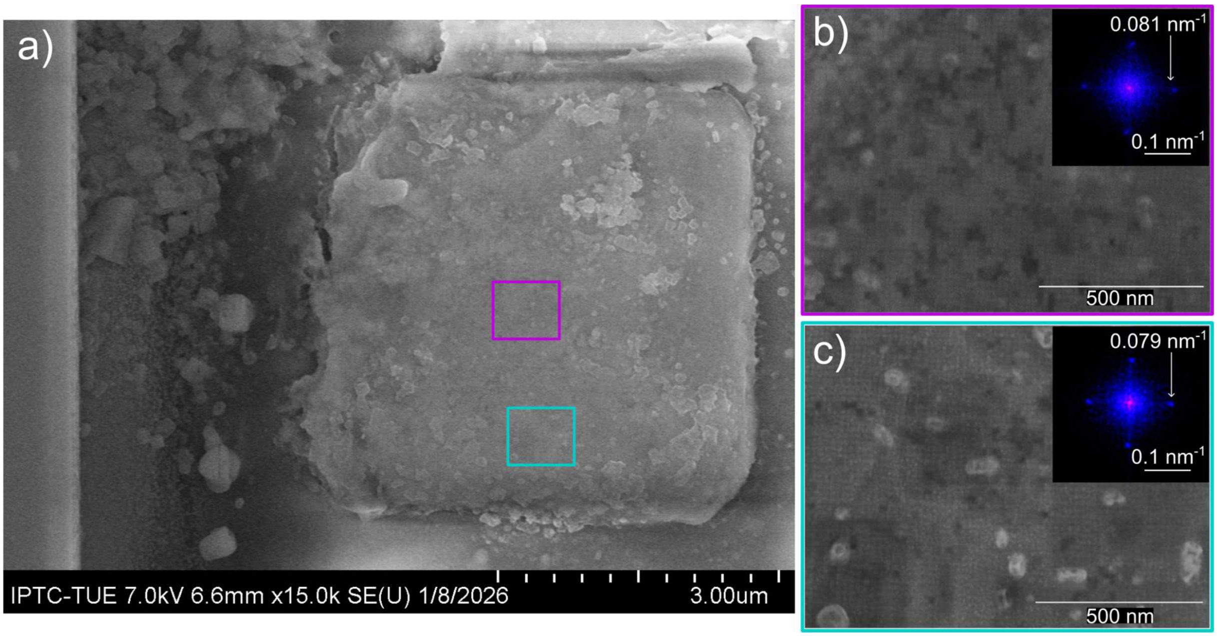

Figure S12: SEM analysis of a $CsPbBr_{2.4}Cl_{0.6}$ SL after exposure to an external electric field. a) Image showing the morphology of the whole SL. b) HR SEM image and corresponding fast Fourier transform of the NCs located at the SL center. c) HR SEM image and corresponding fast Fourier transform of the NCs at the center-bottom region.

The negligible differences between **Figures S11a-b** and the absence of noticeable changes in NC order in **Figures S12b-c** indicate that the $CsPbBr_{2.4}Cl_{0.6}$ SLs and their constituent NCs are not degraded by the strong external electric field but remain a well-ordered assembly.

References

(1) Schedel, C.; Strauß, F.; Kohlschreiber, P.; Geladari, O.; Meixner, A. J.; Scheele, M. Substrate Effects on the Speed Limiting Factor of WSe_2 Photodetectors. *Phys. Chem. Chem. Phys.* **2022**, *24* (41), 25383–25390. https://doi.org/10.1039/D2CP03364J.

(2) Dutta, A.; Behera, R. K.; Pal, P.; Baitalik, S.; Pradhan, N. Near-Unity Photoluminescence Quantum Efficiency for All $CsPbX_3$ (X=Cl, Br, and I) Perovskite Nanocrystals: A Generic Synthesis Approach. *Angew. Chem. Int. Ed.* **2019**, *58* (17), 5552–5556. https://doi.org/10.1002/anie.201900374.

(3) Cottingham, P.; Brutchey, R. L. Depressed Phase Transitions and Thermally Persistent Local Distortions in $CsPbBr_3$ Quantum Dots. *Chem. Mater.* **2018**, *30* (19), 6711–6716. https://doi.org/10.1021/acs.chemmater.8b02295.

(4) Hutton, J.; Nelmes, R. J.; Meyer, G. M.; Eiriksson, V. R. High-Resolution Studies of Cubic Perovskites by Elastic Neutron Diffraction: $CsPbCl_3$. *J. Phys. C Solid State Phys.* **1979**, *12* (24), 5393–5410. https://doi.org/10.1088/0022-3719/12/24/011.
